# Artificial Intelligence-Assisted Stimulated Raman Histology: New Frontiers in Vibrational Tissue Imaging

**DOI:** 10.3390/cancers16233917

**Published:** 2024-11-22

**Authors:** Manu Krishnan Krishnan Nambudiri, V. G. Sujadevi, Prabaharan Poornachandran, C. Murali Krishna, Takahiro Kanno, Hemanth Noothalapati

**Affiliations:** 1 Centre for Internet Studies and Artificial Intelligence, Amrita Vishwa Vidyapeetham, Amritapuri 690525, Kerala, India; manukk@am.amrita.edu (M.K.K.N.);; 2 Chilakapati Laboratory, Advanced Centre for Treatment, Research and Education in Cancer (ACTREC), Tata Memorial Centre, Kharghar, Navi Mumbai 410210, Maharashtra, India; 3 Homi Bhabha National Institute, Training School Complex, Mumbai 400094, Maharashtra, India; 4 Department of Oral and Maxillofacial Surgery, Shimane University Faculty of Medicine, Izumo 693-8501, Japan; 5 Department of Biomedical Engineering, Chennai Institute of Technology, Chennai 600069, Tamil Nadu, India; 6 Department of Chemical Engineering, Indian Institute of Technology Hyderabad, Kandi, Sangareddy 502285, Telangana, India; 7 Faculty of Life and Environmental Sciences, Shimane University, Matsue 690-8504, Japan

**Keywords:** stimulated Raman histology, AI-assisted pathology, label-free histology, intraoperative histology, surgical margins, cancer diagnosis

## Abstract

Frozen section biopsy, introduced in the early 1900s, still remains the gold standard methodology for rapid histologic evaluations. Although a valuable tool, it is labor-, time-, and cost-intensive. Other challenges include visual and diagnostic variability, which may complicate interpretation and potentially compromise the quality of clinical decisions. Raman spectroscopy, with its high specificity and non-invasive nature, can be an effective tool for dependable and quick histopathology. The most promising modality in this context is stimulated Raman histology (SRH), a label-free, non-linear optical process which generates conventional H&E-like images in short time frames. SRH overcomes limitations of conventional Raman scattering by leveraging the qualities of stimulated Raman scattering (SRS), wherein the energy gets transferred from a high-power pump beam to a probe beam, resulting in high-energy, high-intensity scattering. SRH’s high resolution and non-requirement of preprocessing steps make it particularly suitable when it comes to intrasurgical histology. Combining SRH with artificial intelligence (AI) can lead to greater precision and less reliance on manual interpretation, potentially easing the burden of the overburdened global histopathology workforce. We review the recent applications and advances in SRH and how it is tapping into AI to evolve as a revolutionary tool for rapid histologic analysis.

## Simple Summary

Although conventional frozen section biopsy is a valuable tool, it can be time-consuming, expensive, and its interpretation largely depends on the expertise of the pathologist. This review discusses a new technology called stimulated Raman histology (SRH), which creates detailed images of tissue rapidly and without the need for specialized dyes. SRH offers high precision and clarity, making it particularly useful in surgical settings. When paired with artificial intelligence, this method can improve accuracy and reduce the workload for pathologists. Through this narrative review, we aim to demonstrate how SRH has transformed rapid tissue analysis, potentially leading to improved clinical decisions and outcomes for patients.

## 1. Introduction

Histopathology, a diagnostic methodology that evolved into a medical discipline by early 1900s [1,2] is still used worldwide as the gold standard methodology for diagnosis, drawing critical insights into the nature and extent of various diseases [3]. In a general sense, the methodology can be explained as the study of changes within tissue that are associated with a disorder or disease. The procedure usually follows a series of critically and precisely drafted steps, of which the major ones are fixation, sectioning, staining and microscopic examination [4,5]. Histopathology offers several advantages, such as being economic, delivering overview about disease pathogenesis, and facilitating early disease detection. Despite these advantages, the modality is hindered by certain limitations, such as diagnostic variability due to manual assessments, its laborious nature, excessive time consumption, a global shortfall of expert pathologists, and limitations in terms of spatial resolution, information about underlying molecular mechanisms, and scarce quantifiability of features [6,7,8]. With the exponential rise in the incidence of life-threatening diseases such as cancer globally [9], existing methodologies often fall short of contemporary demands. This creates an urgent need for a methodology that is reliable, fast and capable of self-analysis. Such advancements could accelerate diagnoses, ultimately aiding millions worldwide by facilitating early and precise disease detection. Vibrational spectroscopy is an extremely promising alternative in this regard.

Vibrational spectroscopy: The principles of quantum mechanics perceive atoms and molecules to be possessing characteristic inherent energy that is constantly manifested as tiny intrinsic vibrations, the frequency of which is decided by the specific type and local niche around the chemical bonds involved. These molecular vibrations are highly specific to the characteristics of the molecules, the analysis of which can yield highly specific molecular information about the sample, which forms the foundational concept behind all vibrational spectroscopic modalities. Typically, a vibrational spectroscopic modality like Raman spectroscopy or infrared spectroscopy measures specific molecular vibrations in the form of a characteristic spectrum [10]. Such a resultant spectrum from a tissue, when recorded and analyzed properly, can yield valuable information about a disease state. While infrared spectroscopy (IR) can provide valuable information about molecular structure and composition, its utility in biological tissue analysis is limited due to many factors, the preliminary one being intense bands corresponding to water, that obscure disease-related biomolecular signatures. Additional challenges include its limitations in effectively representing highly complex systems such as biological tissues and its dependence on extensive sample preparation, suboptimal molecular specificity, and spatial resolution [11,12,13,14,15]. Consequently, Raman spectroscopy is often the preferred choice due to its greater practicality and diagnostic potential.

Raman spectroscopy, and later microscopy, has evolved through various developmental stages since its inception in 1928 by Sir C.V. Raman and K.S Krishnan [16]. It has now long been established as a dependable analytic methodology in mainstream research. The modality is also laden with some limitations, such as its weak signal intensity and inability to characterize large areas in viable time limits, preventing its efficient translation into clinical applications [17,18,19]. On the other hand, it offers specific advantages, such as label-free analysis, being unaffected in the presence of water, minimal sample preparation requirements, high spectral resolution, and its capability to record complex analytes. [20,21,22]. Raman spectroscopy can be broadly classified into linear (spontaneous) [23,24] and non-linear Raman spectroscopy [25,26], with unique principles and applications. While spontaneous Raman scattering is largely about inelastic scattering of photons, non-linear Raman scattering occurs when multiple photons interact with a sample, at times also producing other non-linear effects, such as second-harmonic generation (SHG) [27,28] or third-harmonic generation (THG) [29,30], which often can be recorded and combined to attain enhanced molecular and morphologic information about the analyte [31,32]. Indeed, spontaneous Raman spectroscopy has been utilized extensively for biomedical applications, from label-free imaging of single cells and tissues [33,34,35,36] to disease diagnosis and prognosis [37,38,39,40]; however, it is slow and suffers from low sensitivity. On the other hand, non-linear Raman scattering delivers the advantages of enhanced signal intensity, minimal photo damage to the tissue analyte, high sensitivity to specific molecular vibrations, swift analysis, sub-micron spatial resolutions, and deep tissue analysis [41,42,43]. These features deem it an extremely efficient diagnostic modality. Two prominent versions of non-linear Raman scattering are coherent anti-Stokes Raman scattering (CARS) and SRS. SRS output spectra are more quantifiable as they have a non-resonant background and are simpler to interpret, possessing a higher sensitivity to trace molecules. They are therefore generally preferred over CARS as a diagnostic modality [44].

To trace the development of the SRS, the advent of the laser in 1960 catapulted advancements in non-linear optics, leading to discoveries such as second and third harmonic generation and SRS. In 1962, researchers identified SRS when a ruby laser with a nitrobenzene Kerr shutter produced unexpected emission lines corresponding to Raman-active vibrations. This discovery, along with subsequent theoretical frameworks, led to the clarification that the SRS phenomenon can shift the interacting laser frequencies through Raman transitions. Unlike fluorescence, SRS does not involve energy storage in excited electronic states. Inspired by the similarity to stimulated emission in laser cavities, this phenomenon was termed ‘stimulated Raman scattering’.

In SRS, the excitation laser beam at frequency ω_p_ generates frequency-reduced radiation, known as Stokes radiation ω_s_, through spontaneous Raman scattering. Some of the Stokes radiation beam re-enters the medium while traveling through the cavity, thus stimulating the creation of more Stokes photons and finally producing a coherent beam at the frequency ω_s_. When both these beams coincide at the specimen, at a resultant frequency equal to the difference between their frequencies, and this matches the molecular vibrational frequency, Raman scattering materialises through stimulated excitation. As a result, the Stokes intensity increases by an amount matching the loss from the fundamental laser beam. Usually, the SRL signal is transformed to pixel intensity via electronics and software, and the sample is spatially scanned with spatiotemporally overlapping beams to image a sample surface [45,46,47].

SRS progressed further through developments in the field of laser sources and light-sensing technologies. The advent of femtosecond and later picosecond laser pulses, led to first time-resolved studies on molecular vibration. Researchers recognized that the Raman free induction decay observed in the time domain was directly linked to the Raman line width in the spectral domain, and selective excitation of inhomogeneously broadened bands offered new insights into the mechanisms contributing to line width. In the early 2000s, a new development in SRS led to the use of femtosecond SRS and picosecond lasers as a contrast mechanism in microscopy. In 2008, researchers successfully applied SRS microscopy to bio-image using a pair of picosecond lasers combined with Stokes pulses, unveiling the scope of rapid, high-resolution, label-free, microscopic imaging of unprocessed tissue specimens [45]. Leveraging further technological advancements in the sector, such as fiber-laser technology, the first commercial clinical SRH device, the NIO Laser imaging system, was introduced by Invenio imaging (Invenio Imaging, Inc., Santa Clara, CA, USA), hereafter referred to as ‘NIO system’ in this article. Another notable technology advancement in the device is the incorporation of improved noise cancellation electronics, such as balanced detection-based noise cancellation, which addressed the high noise level usually associated with the fiber-laser technology [48].

Lee et al. have elaborately discussed the advances in the application of SRS in histopathology in the context of intraoperative settings, analysing new updates in instrumentation and computer-aided diagnosis [49]. Their work further extends into utilizing other non-linear modalities that can be exploited to attain additional diagnostic contrast, leading to enhanced histopathology. Li et al. reviewed SRS microscopic techniques and applications in biosciences [50]. Their work in general reviewed applications of SRS microscopy in cell metabolism, neuroscience, tumor diagnosis, drug tracking, etc., combined with various bio-orthogonal Raman tags. Despite the current body of literature, there is especially little reference available on the latest trends in AI-led developments in the field of SRS imaging and on how their combination jointly contributes to superior performance in attaining histopathology results that cater to the requirements of the contemporary global health sector. Therefore, this review covers significant advancements in artificial intelligence (AI)-assisted stimulated Raman histology (SRH) over the past five years, specifically focusing on diagnostic purposes.

## 2. SRH as a Diagnostic Imaging Modality

SRS histology is a rapid, non-destructive, label-free molecular imaging modality that delivers sub-micron-level spatially resolved histologic images. The image contrast corresponds to the molecular vibrational properties of chemical bonds and the concentration of macromolecules (lipids, proteins, and nucleic acids) of the tissue/cell sample [51,52,53]. Unlike spontaneous Raman spectroscopy, SRS is a non-linear optical process, where two spatiotemporally overlapped pulsed lasers synchronously illuminate the sample to coherently excite the selected molecular vibration. The pump beam serves as one laser and the other is a stokes beam, with the frequency difference between the two carefully set to match the vibrational frequency of the chemical bond of interest. The Stokes Raman transitions of the analyte impart a net attenuation of the pump line and a net gain in the stokes line, which can be explained by coupled wave equations where the beams are coupled parametrically by the polarization response of the analyte [54,55,56,57]. By attaining a quantum stimulation of photons transferred via the Stokes beam, in addition to the weak basal scattering imparted from the other laser beam, the overall scattering gets enhanced drastically. In the context of SRS microscopy, the spatiotemporally overlapped lasers are confined to a tight focus point, generating an enhancement of up to 10^8^ times and thus yielding remarkably high sensitivity at ultra-high speed [58].

The potential of SRS for molecular imaging, beyond spectroscopy, was first demonstrated in 2007 on polystyrene beads. This was aided by an instrumentation panel comprising a femtosecond amplifier laser source and photodiode array [59]. Yet, clinical translation remained a challenge due to the technological requirements being highly demanding and the lack of optics/hardware options that were available at that point in time. For example, the basis of SRS microscopic methodology consisted of the two laser pulsed trains that needed to temporally overlap by less than the pulse duration (<100 fs) and within a spatial range smaller than the focal spot size (<100 nm). It demanded advanced solid-state lasers equipped with continuous water cooling, all of which posed stiff challenges in its clinical translation. In 2008, Freudiger et al. [45] developed a microscopic system for SRS-based three-dimensional multiphoton vibrational imaging, which reported higher sensitivity achieved by means of high-frequency (megahertz) phase-sensitive detection. The study showcased a range of biomedical applications, like discerning distributions of omega-3 fatty acids and saturated lipids in living cells, imaging of brain and skin tissues based on intrinsic lipid contrast, and monitoring drug delivery across the epidermis. Saar et al. enabled in vivo SRS imaging by using a microscopy arrangement that markedly enhanced the collection of backscattered signals and increased imaging speed by three orders of magnitude, achieving video-rate capabilities [60]. This work was a breakthrough that allowed the potential of SRS-mediated quick label-free in vivo imaging to be established. Furthermore, Ozeki et al. demonstrated that tissues can be imaged using SRS at the rate of 30 frames/second with frame-by-frame wavelength tunability [61]. The biggest relevance of the work is the multicolor profiling of different constituents, whereby spectral images were analyzed using independent component analysis, which could detect even minute variations in spectral features and impart colors based on them. Ji et al. showcased that SRS could reveal human brain tumor infiltration in unprocessed surgical specimens from 22 neurosurgical patients, attaining near-perfect agreement with H&E light microscopy (k = 0.86) [62]. To facilitate the use of SRS data in brain tumor surgeries without requiring expert interpretation, the team developed a classifier that attained 97.5% sensitivity and 98.5% specificity in detecting tumor infiltration. Lu et al. also demonstrated the capabilities of SRS using a microscopic system to deliver pathology-like information based on molecular contrasts and high-level sensitivity [63].

It was in 2017 that Orringer et al. established the utility of SRS microscopy for intraoperative histology by capitalising on the recent developments in fiber laser technology, marking the beginning of a new era for Raman spectroscopy in clinical aspects that eventually led to the development of the first clinical SRS microscope [48]. Basically, the NIO imaging system processes freshly excised surgical tissues through three steps: (a) image acquisition (approximately 2 min) (b) image processing (approximately 10 s) and (c) image diagnosis (approximately 20 s). The system generates H&E-like SRH images by mapping the major Raman shifts (corresponding to CH_2_ and CH_3_) from each specimen within 2.5 min for a sample area of 1 mm^2^. The NIO system can function as an effective, streamlined substitute to conventional histologic methods that can save transferring specimens out of the operating theatre to the pathologic facility for processes like sectioning, mounting, staining and pathologic evaluation. The methodology can also help preserve key tissue architectural features for analysis, which may otherwise get lost during the pre-processing steps of conventional frozen sections. The imaging process of the methodology is followed by heatmapping in a manner to match with the H&E images. The feature that SRH imaging is actually a high spatial resolution profile of molecules of interest (molecular fingerprinting) is an advantage when assessing complex structures such as biological tissues with high dependability.

Speaking of limitations, as in any other optical modality, tissue penetration depth is a concern in SRH too, restricting its applications on thicker tissues. This may necessitate careful and precise management of tissue surfaces to be profiled. Furthermore, the high-intensity laser lights used in the methodology can lead to phototoxic effects, affecting the Raman signal output over time owing to photobleaching, as well as some extent of alteration in analyte physiology, like that of cells, which should also be addressed during the analysis. Additionally, for better practicality, the imaging is mainly based on the contrast between Raman peaks corresponding to lipids and proteins. In rare cases, this can lead to suboptimal profiling of the analyte surface, especially in the context of broader organ applications. This issue is being addressed by engaging the AI methodology that considers even the minutest differences in peaks, such as intensity, spatial patterns, and peak ratio analysis. However, beyond all these limitations, it offers strong advantages that render it an effective solution for an automated, standardized method for intraoperative histopathology.

**Figure 1:**
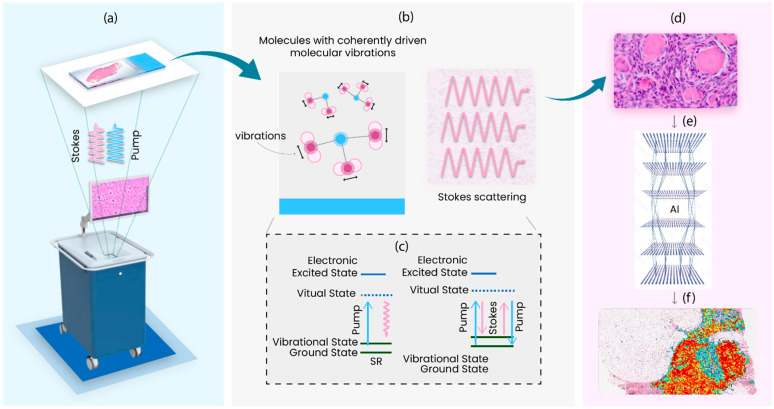
Overview of SRH workflow. (**a**) The tumor specimen obtained intra operatively is loaded onto slides and SRH imaging is performed. Stokes and pump lasers illuminate the sample and (**b**) induced molecular vibrations within the sample. The laser excitation causes energy transitions as shown in (**c**). The molecular perturbations produce coherent Raman scattered photons that will be collected and pseudo-colored to generate stimulated Raman histology images, as shown in (**d**). In (**e**), the resultant images are processed using advanced AI modalities to identify regions of different pathologic features which are heat mapped, as in (**f**), for easy processing by pathologists.

As illustrated in Figure 1, the SRH process begins by obtaining the tissue specimen during surgery and placing it on slides for imaging. Two spatiotemporally synchronized laser beams—the Stokes line and pump line lasers—illuminate the sample. These lasers are tuned to specific frequencies corresponding to molecular vibrations of interest, typically focusing on lipid and protein Raman peaks. The pump laser excites the sample’s molecules to a higher energy state, while the Stokes laser induces specific molecular vibrations. The energy disparity between the pump and Stokes photons enhances specific Raman peaks.

The scattered photons are collected, mapped, and processed to create molecular images of the tissue slices. AI algorithms further analyze this image, with outputs heat-mapped to aid pathologists and healthcare workers in interpretation and identification.

### 2.1. Bone

During sinonasal and skull surgeries, intraoperatively assessing the histology with accuracy and speed is extremely important, which often turns up as a challenge to existing histologic methods. Histology of skull base tumors is complicated by their multiple histologic subtypes and higher rates of positive margins when compared with other anatomical sites [64,65]. Shin et al. used a fast simultaneous two-channel STS imaging technique in combination with a new pseudo-H&E recoloring methodology [66]. Their modularized assessment style extends beyond accuracy in diagnosing cancer by analyzing the degree of agreement between neuropathologists’ confidence in SRH images, H&E-stained frozen and formalin-fixed, paraffin-embedded (FFPE) tissue sections. The results reveal that SRH is effective for establishing a diagnosis using fresh tissue in most cases, with 87% accuracy relative to H&E-stained FFPE sections. However, the authors opined that low stromal lipid concentration always poses stiff challenges for pseudo-coloring, as the process is heavily dependent on lipid/protein contrast, and is therefore not equally efficient for all types of tumors. In another reported work, Jiang et al. utilized SRH to image skull base tumors in patients using an NIO System [67]. They used a CNN architecture implementing three representation learning strategies; cross-entropy, self-supervised contrastive learning and supervised contrastive learning. The cross-entropy strategy yielded an overall diagnostic accuracy of 91.5%, while self-supervised contrastive learning and supervised contrastive learning achieved 83.9% and 96.6%, respectively. Additionally, the trained model successfully delineated tumor-normal margins, and identified and detected regions of microscopic tumor infiltration in meningioma SRH images. Recently, Fitzgerald et al. evaluated the usability of SRH in combination with color-matching algorithms in order to generate images resembling those FFPE sections [68]. The study also used NIO system for SRH imaging. The work reported a significantly faster median analysis time of 4.3 min (compared to 44 min in the frozen section), as well as sensitivity, specificity, precision, and overall accuracy of 93.3%, 94.1%, 93.8%, and 93.3%, respectively. A concordance of (Cohen’s kappa (κ) = 0.89) was seen between the SRH and frozen sections. The authors reported that, while they could effectively detect loss of polarity, high nuclear/cytoplasmic ratio and nuclear pleomorphism, differentiating mitotic figures from apoptotic cells was still challenging.

Musculoskeletal disorders stand as the primary cause of disability on a global scale, and low back pain is the primary factor responsible for disability in over 160 countries [69]. One of the most prominent reasons for lower back pain is lumbar disc herniation, which is a displacement of disc material beyond the space between intervertebral discs, with a high incidence rate of 5 to 20 cases per 1000 adults annually [70]. Percutaneous endoscopic lumbar discectomy (PELD) is an effectual treatment for lumbar disc herniation, and the process mandates swift histological identification of dissected tissue to steer the process. During PELD, fibrotic adhesion is observed between the nerve root and surrounding structures, which needs to be clearly demarcated. Zou et al. demonstrated the applicability of SRH to profiling lipid and protein distributions in combination with SHG and TPEF, which were utilized to image elastin and collagen fibers, using a home-built microscope system [71]. During PELD procedure, fibrotic adhesion between the dura (nerve root) and the surrounding peridural soft tissue structures, such as intervertebral disc (IVD) and ligamentum flavum (LF), can be observed and it is necessary to distinguish between them. Therefore, they measured SRS images at the CH_2_ (2845 cm^−1^) and CH_3_ (2930 cm^−1^) channels of the nerve root to understand the distributions of lipids and proteins. The nerve roots showcased SRS spectral characteristics more like those of lipids, whereas IVD and LF shared similar SRS profiles to proteins. The study also showed significant differences in the non-linear optical characteristics between the dura and surrounding soft tissues, demonstrating the potential for intraoperative differentiation of diverse types of peridural soft tissues to enhance surgical outcomes of PELD.

In a different work involving spinal cord injuries, Wu et al. performed in vivo spinal cord imaging without introducing immunological artifacts in mouse models, to gain crucial understanding of its pathology and treatment options [72]. The study created a less invasive intervertebral access point by preserving the ligamentum flavum to safeguard the spinal cord underneath, thereby lowering the likelihood of triggering microglia. The study presented an optical clearing technique, enabling repeated imaging at subcellular resolution using two-photon fluorescence and SRS, all while avoiding any inflammation induction. The study employed a self-developed multimodal NLO microscope system combining SRS, TPEF and SHG modalities to visualize iodixanol, cells and collagens simultaneously. Hyperspectral SRS sweeping mode was employed to obtain SRS spectra of solutions and tissues in fingerprint and C-H regions. The study thus crafted a less invasive intervertebral access point for spinal cord imaging in mice and successfully attained long-term, high-resolution imaging without inducing any inflammation.

### 2.2. Breast

Breast cancer is the most commonly diagnosed cancer among adults and in 95% of countries, breast cancer is the first or second leading cause of female deaths [73]. It is a well-established fact that microcalcifications are an incredibly valid indicator of neoplastic processes in breast cancers. Carbonate content is yet another indicator that correlates with local pathology. Despite the diagnostic and prognostic potential, the morphologic and chemical features of calcifications are poorly understood.

Shin et al. visualized breast calcification chemical compositions at high spatial resolution by utilizing spatially resolved qualitative and quantitative information [74]. The study employed SRS microscopy to examine a range of breast alterations, encompassing benign to neoplastic processes, including atypical ductal hyperplasia, ductal carcinoma in situ and invasive ductal carcinoma. The methodology involved ratiometric analysis used to quantify hydroxyapatite, a main diagnostic feature of cancer. A customized two-color microscopy setup consisting of SRS and SHG was used for the analysis. Results reiterated the already established fact that the average carbonate content tends to decrease with increasing malignancy potential. The work revealed that the microenvironment surrounding neoplastic processes significantly impacts the local carbonate content distribution. Specifically, the carbonate content reduced near the edges of calcifications closest to neoplastic cells indicating an acidified microenvironment as malignant cells proliferate. Additionally, the spatial heterogeneity of carbonate content could potentially be a diagnostic indicator of malignancy. Using SRH, the work reported sensitivity and specificity of 85% and 88%, respectively, when the level of carbonate content level was employed as the sole discriminator. To improve the accuracy in cases of diagnosing fibro-adenoma from invasive ductal carcinoma, SHG was used as the second modality, yielding a significant improvement; it was able to separate the cases with a higher sensitivity and specificity of 94% and 85%, respectively.

Bouzy et al. combined Raman and optical photothermal infrared for the first time (O-PTIR)—a modality that can simultaneously record IR and Raman spectra from a single point—to study microcalcifications by analyzing molecular composition at identical locations with high spatial resolution [75]. They employed a mIRage IR microscope (Photothermal Spectroscopy Corp, Santa Barbara, CA, USA) and also utilized other multiphoton imaging techniques to validate the existence of carbonate ions within the microcalcifications. The multiphoton setup, including the SRS and SHG methodology, was facilitated using an experimental setup customized onto a confocal inverted microscope. The tissue unveils the diversity within the breast microcalcifications (BMCs) using the O-PTIR system, with the limitations being low field of vision and high acquisition times. The SRS and SHG techniques were further employed for probing the collagen content of the calcifications. Overall, this multimodal approach constructed SRS images to precisely mimic H&E sections. The work established a means to analyze microcalcifications by iteratively refining the area of interest.

Ni et al. developed a two-color SRH, a modality that generated a high-content SRH (HC-SRH) system that delivers both morphological and chemical data for diagnosing breast cancer [76]. The methodology could successfully profile unsaturated lipids, extracellular matrix, cellular protein, saturated lipids and water in the breast tissue, which could in turn map the duct, fat cell, stroma, vessel and necrosis. In contrast to the well-developed two-color SRH, HC-SRH delivers extra chemical information aiding cancer diagnosis. The notable advantages are that selective spectral sampling enhances HC-SRH speed by one order of magnitude, while also delivering outstanding contrast for diverse breast tissue components. To analyze the clinical applicability of the methodology, a fiber-optical parametric oscillator (FOPO)-based HC-SRH system was developed instead of a solid-state system. Leveraging the rapid, widely tuning capability of the FOPO, the spectral coverage of the HC-SRH was extended to the fingerprint window, delivering extra contrast for nucleic acid, solid-state fat and amino acids in breast tissue. The resulting two-colored images provided a deeper understanding of the subtle chemical changes associated with cancer progression.

### 2.3. Live Cell Imaging

Live cell imaging is a very valuable tool in the context of biological and biomedical applications including disease diagnosis. It helps cell biologists to learn valuable clues about the structure and functions of the cells and tissues, which in turn largely helps the diagnosis. Various advancements in technology are tremendously helping the live cell imaging process. However, the methodology is still challenging when it comes to maintaining the cells in a healthy state under the optical devices for extended periods of time. SRH is a methodology that is capable of imaging cells with high spatio-temporal resolution over extended periods of time, without causing any damage to the cells.

To tap into this potential of SRH, Yuan et al. designed and fabricated a flexible chamber for time-lapse live-cell imaging where the detection of SRS signal is performed via an upright microscope frame [77]. The developed enclosure and chamber can be incorporated into a conventional SRS imaging system. The enclosure is designed to contain and maintain the SRS microscope and the whole imaging environment at 37 °C. Results show that the temperature within the flexible chamber reached the anticipated temperature within 1.5 h, and it remained stable for at least 24 h, indicating high suitability for cell-based studies. The authors report that temperature instability induced focal drifts and subtle vibrations during the recording process that should be addressed for a better SRS recording.

In yet another reported work, Liu et al. analyzed the effects at the biological and molecular levels, leading to the anti-survival effects on mantle cell lymphoma (MCL), as a comparison between first- and second-generation BTK inhibitors (BTKi) [78]. The biological impact of BTKi on MCL cell chemotaxis and lipid droplet accumulation was examined in three different MCL cell lines using transwell and SRS imaging analysis, respectively. SRS imaging was performed using a femtosecond SRS microscope with the laser frequency tuned to a C-H stretching vibration band at 2845 cm^−1^, without any cell damage. Quantitative examination of lipogenesis at single-cell level via SRS imaging unveiled that BTKi treatment notably decreased lipid droplets accumulations in MCL. The overall effects of BTK inhibitors can be summarized as inducing apoptosis while suppressing chemotaxis and lipid accumulation.

The peritoneum is a serous membrane that lines the abdominal cavity, and its lavage cytology is the established methodology used to intraoperatively diagnose peritoneal metastasis (PM), a prevalent type of distant metastasis. Xun Chen et al. addressed the issue of low sensitivity (<60%) in peritoneal lavage cytology using a customized three-color SRS microscope [79]. This study identified twelve morphological single-cell features and a compositional difference between PM cells and non-PM cells, which includes lipid protein ratio, cellular area, etc. The work further crafted a phenotyping algorithm for single cells to further transform the identified raw features into feature matrix. In comparison with histopathology (gold standard), the SRH methodology attained a sensitivity and specificity of 81.5% and 84.9%, respectively, with an AUC of 0.85. The whole procedure was performed within a span of 20 min for each patient.

In the case of prostate cancers, focal therapy (FT) is an approach where clinically significant prostate cancer is removed and nearby normal areas are preserved, thereby minimizing treatment-related toxicity. This procedure mandates the need for quick sampling by core needle biopsy based rapid histology to precisely demarcate the cancer-affected area where SRH is considered a strong alternative methodology. Ao et al. applied SRH and CNN in this context [80]. An external test dataset validated the CNN’s performance with an accuracy of 84.4%. This work further calculated Gleason scores from 21 cases of core needle biopsies; the deep learning SRS system showed an accuracy of 71% compared to gradings from three pathologists, establishing the promise of deep learning-assisted SRS platforms in assessing the grade of prostate cancer tumors. This work went ahead to employ a diagnostic CNN trained on images from 61 patients that classified Gleason patterns of prostate cancer, reporting an accuracy of 85.7%. This methodology suggests provision for rapid histopathology and automated Gleason scoring without complex processing.

Zhang et al. proposed an automatic cell-counting model for SRS images. Cell counting of actual human brain tumor specimens was conducted [81]. The research established the capability of the methodology to decrease whole-brain imaging time from 70 min to just 8 min. The major challenges to high-speed imaging are the imaging rate of multi-color SRS and the efficiency attained in image stitching. To attain speed, the study incorporated parallel dual-phase SRS detection with strip mosaicking. The dual-phase SRS Stokes beam is split into two beams, each probing specific target Raman frequencies that are modulated with a 90° phase difference. Hence, the SRS signals at two specific Raman frequencies are generated as in-phase and in-quadrature components. The respective time delays are programmed to probe high-frequency Raman modes corresponding to (2845 cm^−1^) and (2930 cm^−1^), allowing simultaneous imaging of lipid and protein contents with minimal crosstalk, allowing faster imaging. The study also used a strip mosaicking methodology, where a focused laser in line-scan mode is used, with the sample moving at a constant speed perpendicular to the laser line. This methodology delivers comparable imaging results to the tiling method, but with improved speed. The study yielded results with an AUC above 98% and an R value of 0.97 for cell counting correlation between SRS and H&E-stained histological images.

Core-needle biopsy (CNB) is an initial diagnostic approach for breast cancer. Nevertheless, the intricacies of tissue processing and worldwide scarcity of pathologists frequently present hurdles to swift diagnosis of fresh biopsies. The work done by Yang et al. compared the results of SRS imaging with gold standard H&E staining on adjacent frozen tissue sections, using a home-built non-linear optical microscope [82]. Utilizing SRS, the fresh, unprocessed biopsy tissues underwent imaging. A weakly-supervised learning approach, namely the multi-instance learning (MIL) model, was employed to differentiate between benign and malignant cases, and was subsequently compared with the performance of supervised learning model. Additionally, gradient-weighted class activation mapping (Grad-CAM) and semantic segmentation were employed to spatially delineate benign/malignant areas. Results indicated that the MIL model could achieve superior classification performance compared to supervised learning, attaining diagnostic accuracy of 95% on 61 biopsy specimens. Grad-CAM facilitated the visualization of histological heterogeneity within the CNB by the trained machine learning (ML) model.

### 2.4. Gastric

Gastric cancer ranks as the fifth most diagnosed cancer and the third primary contributor to cancer-related fatalities globally. Surgical resection and lymphadenectomy are the only potentially curative treatment approaches for gastric cancer. There is a serious need for new modalities that can rapidly image and diagnose tissues, especially in an intraoperative evaluation and gastroscopy context. Sarri et al. developed a framework based on SRH and SHG to render images of colon and pancreas sections, both frozen and fresh, in alignment with conventional H&E staining-based techniques for healthy, precancerous and cancerous colon and pancreas tissue sections [83]. Additionally, a new rapid SRH imaging method was devised, capturing all essential information at the pixel level to generate instantaneous SRH images. These results were attained using a customized instrument which could perform a variety of non-linear, label-free imaging modes, employing a custom-built three-color setup, which was obtained from elsewhere. The two modalities selected for tissue imaging were ƛ-switch and frequency-modulated SRS, which were combined with SHG- and TPEF-generated SRH images (H&E-staining quality at pixel level), eliminating the need for sequential acquisitions and making the whole process instantaneous. The reported time requirement for scanning 100 μm × 100 μm (200 px × 200 px with dwelling time of 40 μs/px and 3 accumulations/pixel) was 9 s, making the imaging time for a 1 mm × 1 mm stitched image ~25 min. Although the recorded time is unable to challenge the speed of the intraoperative quick staining method, the image quality of the SRH output can be compared to full H&E staining, which takes anywhere from 24 h to 72 h to produce. Another notable advantage is that the images remain unaffected by the subtle tissue movements during the analysis, as signals are acquired simultaneously.

In yet another work, Sarri et al. compared the consistency of the alignment between SRH and H&E images on identical cryogenic slides for both normal and cancerous tissues [84]. The same instrumentation as reported in the previous study was used for image acquisition, combining SRS and SHG modalities. The fresh colon and pancreas tissues obtained from tissue resection surgery were assessed. The samples were imaged with SRS and SHG, and two slides from the same sample were stained with toluidine blue and HES for comparison with SRH. SRS, coherent anti-Stokes Raman scattering (CARS), second harmonic generation (SHG), and two-photon excited fluorescence (TPEF) imaging were performed using a custom-built three-color setup. Tissues were examined by trained histopathologists at both microscopic and macroscopic scales to perform accurate medical diagnosis. The Raman peaks corresponding to 2845 cm^−1^ (CH_2_ bonds) and 2930 cm^−1^ (CH_3_ bonds) were identified and nuclei images generated by simple subtraction. The study established quasi-faultless complete matching between SRH and H&E-stained images. It also substantiated the ability of SRH imaging to rapidly determine critical instances usually encountered intraoperatively, such as detecting peritoneal metastasis in the omentum, which played a decisive role in the workflow of the surgery. The work also showed that SRH could produce results comparable to H&E against thick tissue biopsies too (obtained directly after excision surgery). General architecture variations of the tissue and structural shifts at the subcellular level could be assessed by SRH microscopy on millimeter-sized GI tract tissues.

Conventional SRS necessitates adjusting picosecond lasers to achieve considerable chemical specificity, albeit at the expense of the speed of analysis, which can be a downside in instances like that of gastroscopy, where speed of analysis is also critical. A study by Liu et al. demonstrated that single-shot femtosecond SRS delivers maximum speed and sensitivity and preserves the chemical resolution by using U-Net [85]. The femtosecond-U-Net combination enabled real-time pseudocoloring, attaining rapid SRH imaging of a ~2 × 2 mm^2^ tissue, which highly agreed with the findings of standard H&E. The isolated flavors of the chemical contrast deliver important histological details that can help to attain high diagnostic accuracy. The optical simplicity, speed and stability of the developed method makes it highly favorable for clinical translation. The AI part of the work is discussed in detail in Section 3. Furthermore, they also developed a diagnostic neural network (CNN) with data from 279 patients that eventually diagnosed gastric cancer with an accuracy of >96%. The study demonstrated the possibility of SRH to be used as a valuable tool for automated intraoperative diagnosis.

### 2.5. Gout

Gout is a prevalent type of inflammatory arthritis that is extremely painful. It is characterized by deposition of monosodium urate (MSU) monohydrate crystals in the tissues, which can be rapidly detected by using SRS microscopy in a label-free manner [86]. Zhang et al. first tested SRS and SHG methods using rat models, simultaneously analyzing MSU, checking its diagnostic capability, and distinguishing between pseudogout and calcium pyrophosphate deposition disease (CPDD), acute gout arthritis and comorbidity [87]. A home-built setup with combined SRS and SHG microscopies was used to characterize MSU in crystalline and amorphous forms simultaneously. The study imaged synovial fluid and surgical specimens to obtain the histopathology of MSU deposition. By analyzing MSU depositions in human surgical specimens and rat models, the study enabled early diagnosis of gout and distinguished it from pseudogout based on the unique Raman signatures of MSU and CPPD. SRS analysis unveiled the optical traits of MSU deposition across various pathophysiological stages, aligning closely with corresponding features observed through immunofluorescence histochemistry, thereby confirming its reliability. The MSU peak at 630 cm^−1^ and the corresponding CPPD peak at 1050 cm^−1^ were used to distinguish gout from pseudogout. SRS microscopy has the advantage of background-free detection and enhanced tissue penetration depths. It was also observed that MSU correlates reasonably with the inflammatory cytokine expression levels, consistent with previous studies. These observations can critically establish the capability of the SRS methodology.

### 2.6. Liver

Liver transplantation has turned out to be a lifesaving means and is an established intervention in patients with chronic and acute final-stage liver diseases. Liver transplantation has evolved into a safety net to treat various liver diseases where all other medical interventions have failed. However, the process of liver transplantation is very delicate, and a few factors influencing graft quality can affect the success of the procedure.

Typically, graft quality for transplantation is evaluated by visual inspection, which heavily depends on the surgeon’s expertise and is thus prone to high variability. Ember et al. developed a method for objectively assessing graft tissue quality in real time, non-invasively and quantitatively using SRH [88]. A porcine model-based trial confirmed circulatory death followed by normothermic regional perfusion (NRP), which enables the evaluation of liver quality under three distinct conditions: preceding cardiac arrest, during warm ischemia (WI) and post-NRP. To understand the changes, liver left-median lobe biopsies were acquired prior to circulatory arrest, following 45 min of WI 2 h post-NRP. These were all analyzed using three different methods: spontaneous Raman spectroscopy, SRS and staining. The study utilized three forms of Raman spectroscopy: spontaneous, stimulated and handheld. Conventional spontaneous Raman microspectroscopy was used to analyze tissue sections up close. SRS microspectroscopy was achieved using a Leica microsystem SP8 multiphoton confocal microscope armed with a CARS 1200S filter wheel, which detected signals in a photomultiplier tube detector in transmission mode, acquiring images at 1024 × 1024 resolution. The work made important observations in the context of liver transplantation, such as diminishing blood volume preventing microvascular impairment post-circulatory arrest. The work also established that SRS can be utilized to visualize intact red blood cells (RBCs) at high resolutions.

### 2.7. Neuro

In 2017, Orringer et al. reported groundbreaking work in developing a clinically compatible SRH device for detecting infiltration of brain tumors within human tissue [48]. This work demonstrated the first intra-surgical application of SRS microscopy by using a portable fiber-laser-based microscope, which generated SRS images that matched H&E and revealed vital diagnostic features. This work was soon to be followed by another imaging study on fresh tissue samples gathered from paediatric participants enrolled in advance for brain tumors. Diagnoses based on SRH reported near-perfect diagnostic agreement (Cohen’s kappa, k > 0.90) and an accuracy of 92% to 96% against the gold standard [89]. Neurological tissues are among the most common subjects for SRH methodologies ever since. The methodology found applications in meningiomas, which constitute a substantial proportion of all central nervous system (CNS) tumors and are the most common form of intracranial, extra-axial neoplasms. Surgical resection is the main mode of treatment. However, in lots of instances, such as those arising from the skull base, complete removal is often difficult owing to the proximity to critical anatomic structures. Luther et al. used SRH as a method to delineate tissue boundaries while resecting a recurrent, extensive, atypical spheno-orbital meningioma [90].

Identifying glioma recurrence continuously poses a challenge in contemporary neuro-oncology. Differentiating between glioma tumor recurrence and pseudoprogression is crucial to determining treatment options and prognosis. Hollon et al. employed the combination of SRH and deep neural networks to establish its potential in improving the intrasurgical detection of glioma recurrence [91]. In this study, a fiber-laser-based SRH system (<60 s per 1 × 1 mm^2^) was employed to image 35 patients (cohort) with suspected recurrent gliomas following resection or biopsy. The resultant SRH images served to train a convolutional neural network (CNN) and develop an inference algorithm to detect possible recurrent gliomas. The diagnostic performance was evaluated in a retrospective cohort of 48 patients (from an external validation medical center), achieving an accuracy of 95.8%. This clinical SRH approach could successfully image critical diagnostic features of recurrent glial tumors and treatment-induced histologic changes associated with pseudo-progression. It also identified regions with a high probability of recurrence, enhancing clinicians’ assessments of automated diagnostic results. In another approach, Bae et al. applied epi-detected, spectral-focusing hyperspectral SRS microscopy for rapid, label-free molecular analysis of intra-tumoral heterogeneity in glioblastoma (GBM), achieving sub-micron resolution. This distinctive diagnostic platform consists of distinctive spectral-focusing hyperspectral SRS imaging of GBM tissue specimens, SRS images, and spectrum retrieval through a multivariate curve resolution algorithm. Additionally, a quadratic support vector machine model enabled subtype classification for rapid molecular subtyping of GBMs. Both stain-free SRS histological images and 2D subtype maps were obtained within 20–30 min. While SRS histology assesses demyelination status as a new diagnostic feature, SRS mapping provides insights into the intra-tumoral heterogeneity of GBM tissue specimens [92]. The prediction variance for each of the subtypes when using SRH was within a range from 0.44 to 0.79, signifying considerable intra-tumoral heterogeneity across the tissue samples. It also aligned well with single-cell RNA sequencing data, where the correlation coefficients among tumor cells that matched with the GBM samples varied from 0.2 to 0.7, indicating intra-tumoral heterogeneity that is in turn driven by the tumor microenvironment. The authors propose that diagnostic time could be reduced to mere seconds by using a resonant scanner rather than a Galvano scanner.

Beta-amyloid proteins clump together to form plaques that disrupt cell functions, leading to Alzheimer’s disease. Lochocki et al. compared high-resolution fluorescence imaging (both pre- and post-staining) and spectroscopic techniques (Raman mapping under pre-resonance conditions and SRS of amyloid deposits in snap-frozen AD human brain tissue) [93]. The SRS instrument is an in-house-built picosecond system. Three different methods—spectroscopic imaging, fluorescence and ensuant thioflavin-S staining—were performed on the same tissue slices to render direct indications of plaque location and correlate spectroscopic biomarkers with plaque morphology, and especially to reveal differences between cored and fibrillar plaques. The study eventually identified carotenoids as a unique marker that could differentiate between a cored amyloid plaque area and a non-plaque area without prior knowledge of their location. The observed presence of carotenoids suggests a specific neuroinflammatory response to the accumulation of misfolded proteins.

Ji et al. used multicolor SRS microscopy to visualize amyloid plaques in brain tissue from an Alzheimer’s disease (AD) mouse model [94]. The study demonstrated the technique’s ability to differentiate misfolded proteins from normal ones by detecting a blue shift (~10 cm^−1^) in the amide I SRS spectra. The customized SRS microscope performed imaging in the amide I region at approximately 5 s per frame for 512 × 512-pixel images, using a dwell time of 10 s per pixel. Imaging 40 images took ~4 min. In addition, imaging in the high-frequency CH stretch region was performed at ~1 s per frame with a dwell time of 2 s per pixel. The spatial resolution was ~400 nm laterally and ~2 µm axially, with a spectral resolution of ~8 cm^−1^. Afterwards, the results were evaluated by antibody staining on frozen thin sections and fluorescence imaging of fresh tissues. Both imaging methods successfully visualized normal brain structures, including the cortex, white matter, hippocampus, and dentate gyrus. In regions where amyloid plaques were identified by immunohistochemistry, three-color SRS imaging consistently captured them, showing a direct correlation. An intriguing observation revealed by the three-color SRS was the presence of a lipid-rich halo structure surrounding each plaque, potentially originating from degenerated neurites and myelin sheaths. The spectral shift of the amide I band of Beta sheets facilitated the differentiation of misfolded Aβ from lipids and normal proteins.

Pekmezci et al. used a NIO system to measure glioma margin samples for SRH, histology, and tumor-specific tissue characterization [95]. The work addressed the question of whether SRH can be used to image cancer margins that are generally considered to be less cellular and hence harder to image. Alongside semi-quantitative scoring of the margins by three neuropathologists using morphologic features, the work performed a cellularity count, which, as anticipated, corresponded with the semiquantitative scoring models utilized. The successful use of SRH images to identify margins with minimal tumor presence confirms that SRH imaging offers ample cellular and architectural details, surpassing mere cellularity. Generalized linear mixed models were employed to evaluate agreement. The study found that SRH identified residual tumors in 82 of 167 margin specimens (49%), while IHC substantiated residual tumors in 72 of 128 samples (56%) and H&E confirmed residual tumors in 82 of 169 samples (49%). Interobserver compatibility between all three modalities was confirmed.

Neidert et al. utilized SRH to achieve intraoperative near-real-time histopathological analysis. In the study, a total of 429 SRH images from 108 patients were generated and analyzed using the NIO system. The work demonstrated that the utilization of SRH is feasible and beneficial in intraoperative assessment contexts [96].

The user-friendliness and interpretability of an analytical methodology are important parameters that was assessed for SRH by Straehle et al., who attempted to quantify the neuropathological interpretability of SRH acquired in a routine clinical setting without any specialized training or prior experience [97]. SRS microscopy was performed on 117 pathological tissues obtained from 73 brain-spine tumor surgeries. A neuropathologist who was inexperienced in dealing with SRH interpretation assessed the quality of the images in terms of tumor infiltration and provided diagnoses based on the SRH images. Diagnostic accuracy was subsequently measured by comparing the SRH-based diagnoses to conventional frozen H&E-stained sections, with the definitive neuropathological diagnosis serving as the ground truth. Overall, SRH imaging quality turned out to be rated highly, with only 4.2% of all images marked as inconclusive for detecting tumor cells. The diagnostic accuracy of neuropathological conditions was 87.7% and was comparable to the current standard of fast-frozen H&E-stained sections (87.3 vs. 88.9%, *p* = 0.783). This study demonstrated a strong diagnostic agreement between SRH-based and H&E-stained frozen sections (κ = 0.8), indicating that intraoperative SRH imaging provides necessary diagnostic clarity on par with traditional H&E-stained frozen sections.

Autofluorescence differs in the gray and white matter of a healthy brain, just as it does in different brain regions and tumor types. The relationship between autofluorescence characteristics of the human brain and neoplastic changes at a microscopic level was studied by Furtjes et al. using the combination of two-photon fluorescence and SRH [98]. The study found an increased mean autofluorescence signal in healthy brain tissue, in the gray compared to the white matter and in the cerebrum versus the cerebellum, respectively. The signal intensity of carcinoma metastases, meningiomas, gliomas and pituitary adenomas was notably lower compared to the autofluorescence in the cerebrum and dura, but significantly higher than that in the cerebellum. Conversely, melanoma metastases exhibited a higher fluorescent signal compared to both the cerebrum and cerebellum.

### 2.8. Oral

Oral diseases, while being largely preventable, are turning out to be a major health burden for many countries. They are estimated to affect nearly 3.5 billion people. Oral cancer specifically affects the parts of the mouth, the lip and oropharynx, and it is ranked thirteenth most common cancer globally.

When it comes to laryngeal squamous cell carcinoma, the importance of maximally resecting the tumor while preserving the healthy tissue is high, as is that of intraoperative histology. Zhang et al. utilized deep learning methodologies in SRS microscopy-acquired images to analyze the diagnostic concordance and its classification efficiency when compared with standard histology [99]. The study first analyzed the ability of SRS imaging in detecting characteristic features using a custom-made SRS microscopy arrangement. The study proved that zoom-in SRS images could reveal microscopic features of normal larynges, including intact basal lamina, basal layer and the squamous mucosa layer, as well as clearly differentiate its diagnostic features, including cytological atypia, abnormal arrangement of neoplastic cells and lymphocytes, cancer nests and keratin pearl. Furthermore, the study evaluated the ability of SRS microscopy in intraoperative tissue assessment contexts; they collected 80 SRS and 80 H&E images, and the mix of these images was assessed by three professional laryngeal pathologists. Cells were assessed to be neoplastic or normal based on cytology and histoarchitecture. Statistical analysis of the pathologists’ interpretations of SRS and H&E yielded high concordance, with the Cohen’s kappa value (k) between them ranging between 0.905 and 0.942. Notably, the pathologists were highly accurate in distinguishing between neoplastic and normal larynx tissues, with a Cohen’s kappa, k > 90. The team utilized a deep learning methodology, ResNet34, to differentiate between normal and neoplastic cells; this methodology classified 33 specimens with 100% accuracy and identified tissue neoplasia even in instances where it appeared normal to the naked eye.

Cancer heterogeneity is expressed through multiple aspects, which include evolving genetic changes, molecular variations and morphologic abnormalities of cells in distinct subpopulations. It is helpful to analysz tumor heterogeneity in a precise and comprehensive way. Chen et al. attempted to combine various modalities to fully characterize the genomic and transcriptomic profiles of cells with high spatial resolution to characterize human oral squamous cell carcinoma [100]. To attain this, they combined histological analysis coupled with spatially resolved multiomics analysis in tissue sections, without fixation or staining. This approach employed SRS microscopy to furnish chemical contrast that reveals histological tissue architecture, facilitating high-resolution in situ laser microdissection, which was performed using a home-built SRS system. The system generated 2-color SRS images based on 2850 cm^−1^ and 2950 cm^−1^ Raman shifts. Furthermore, SRH-profiled microtissue samples were processed for DNA/RNA sequencing to determine unique genetic profiles corresponding to distinct anatomical regions. Named as the SRH-SMD methodology, this study demonstrated its capabilities by profiling copy number and gene expression alterations to histologically characterized regions in human oral squamous cell carcinoma (OSCC). This approach enabled the dissection of cancer heterogeneity across multiple measurement modalities encompassing morphology, genome alteration, gene expression and gene fusions.

Steybe et al. used SRH to produce digital histopathologic images of 80 tissue samples from eight OSCC patients [101]. Subsequently, the obtained images were compared with conventional H&E normal mucosa, squamous cell carcinoma, lymphatic tissue, muscle tissue, salivary gland tissue, connective tissue, adipose tissue and inflammatory cells. Cohen’s kappa agreements were calculated between images and sections to analyze the match between two. High correspondence between H&E and SRH (kappa: 0.880) and high accuracy of SRH (sensitivity: 100%; specificity: 90.91%; PPV: 90.00%, NPV: 100%; AUC: 0.954) were showcased in the study. The work concludes that SRH provides high accuracy in discriminating neoplastic and non-neoplastic tissues, while the subclassification results of non-neoplastic tissues in OSCC patients also depend on tissue type. However, all patients involved in the study had a diagnosis of oral squamous cell carcinoma, and the authors perceive that this might have introduced some bias that boosted the sensitivity, specificity and predictive values of the study.

### 2.9. Respiratory

Pure multi-walled carbon nanotubes are anticipated to have very low toxicity in vitro, which was assessed for lung and systemic impacts in mouse trials by Migliaccio et al. [102]. SRS was used to recognize particles in the lungs, kidneys, spleen, liver, mediastinal and brachial lymph nodes, and olfactory bulb. The images were obtained using an in-house-built SRS imaging setup. The imaging was first performed at 2700 cm^−1^ and later at 2930 cm^−1^, which represents the -CH_3_ symmetric stretch, with the assumption being that the first signal originated from the MWCNT (multiwalled carbon nanotubes) due to two-photon absorption and/or thermal response, while the latter signal corresponded to biological tissue. Thus, MWCNT localization was obtained by overlaying it with the tissue SRS image at 2930 cm^−1^. This work advocates the need for extensive assessments of nanomaterial exposures that address both short- and long-term effects.

## 3. AI Based SRH

In a study aimed at implementing a swift, automated analysis of skull base tumors using intraoperative SRH imaging alongside AI, Jiang et al. implemented a ResNet50 architecture, containing 25.6 million trainable parameters, as a feature extractor [67]. The work was designed to probe into the ability of SRH to (a) capture diagnostic features of skull base tumors, (b) use an AI-based computer vision system to effectively identify diagnostic parameters from an SRH image and (c) detect microscopic tumor infiltration in meningioma surgeries. The SRH imaging was conducted utilizing two imaging systems: a prototype clinical SRH microscope, and intraoperative imaging performed using the NIO system). Two Raman regions at 2845 and 2930 cm^−1^ were chosen to feature lipid-rich and cellular locales. The subtracted image of these regions highlighted cellularity and nuclei. A virtual H&E color scheme was applied to transform the raw SRS images into SRH images. Then, a learning methodology called contrastive learning was employed, where the overall objective was to create an embedding space where similar data points are placed close together and dissimilar data points are placed far apart. A contrastive loss function was used to encourage the model to minimize the distance between the representations of similar points while maximizing the distance between dissimilar pairs, based on theoretical aspects. The team hypothesized that contrastive representation learning is more robust to label noise, based on theoretical aspects. In this study, a ResNet50 CNN architecture was used as a feature extractor. The feature extraction model produces a 2048-dimensional feature vector for each input image, which is subsequently reduced to 128 dimensions prior to calculating the cosine similarity metric. The model analyzed three separate loss functions—supervised categorical cross-entropy, self-supervised contrastive, and supervised contrastive—to identify the best performer. In the self-supervised learning setting, a pair of data was generated through transformation processes, such as blurring or flipping, attaining image versions x1 and x2 and then normalized vector representations z1 and z2. This process eventually attains separate clustering of positive and negative vector representations on a unit hypersphere. The contrastive learning models were optimized using stochastic gradient descent, and each model was trained using a batch size of 176 for 4 days on GPUs. Upon completion of training the feature extraction model, the features were classified using a linear classifier layer trained with cross-entropy loss, the Adam optimizer, and a batch size of 64 over 24 h on GPUs to obtain a probability distribution of output classes.

For testing purposes, the whole-slide image was fragmented into 300 × 300-pixel patches. These patches were fed into the trained models to work out the probability distribution. The patch-level probability distributions were summed up to infer the overall slide status using a “soft” aggregation approach as opposed to the “hard” aggregation of the patches. This facilitated the detection of microscopic tumor infiltration, which was proven in a skull base meningioma surgery. The study attained a 5.1% increase in the accuracy of diagnostic classification using supervised contrastive learning compared to models based on cross-entropy. In the multicenter testing set, cross-entropy achieved an overall diagnostic accuracy of 91.5%, self-supervised contrastive learning achieved 83.9%, and supervised contrastive learning achieved 96.6%. The trained model was able to segment tumor-normal margins and detect regions of microscopic tumor infiltration in meningioma SRH images. For segmentation purposes, the team improvised a previously engineered method for segmenting the SRH image by employing patch-level forecasting, which incorporated a local neighborhood of overlapping patch predictions to render a high-resolution probability heatmap, generating a two-channel image with the predicted tumor class as the first channel and the most probable non-tumor class as the second channel.

**Figure 2:**
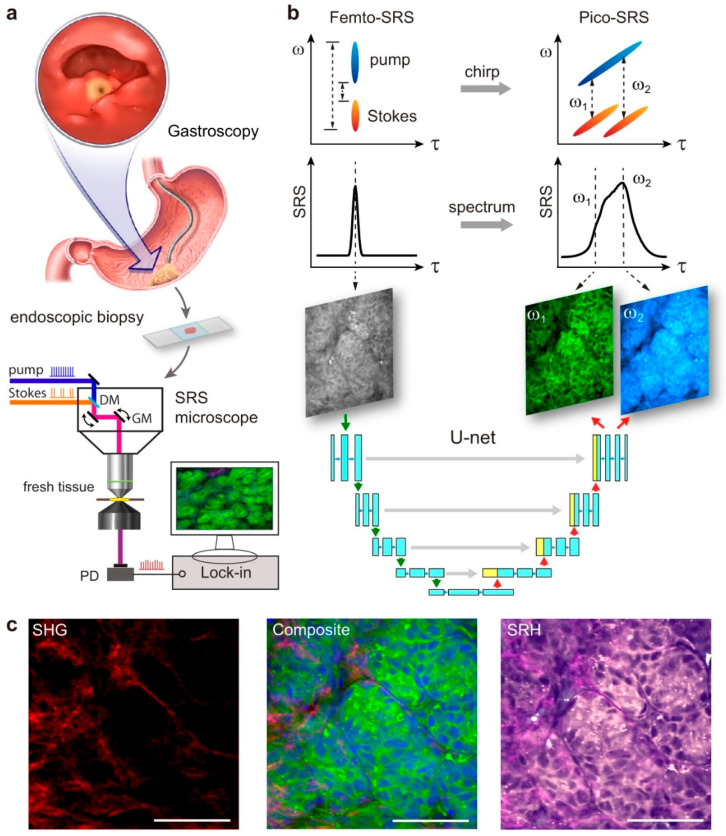
(**a**) The representation portrays the process of gastroscopy and the collection of fresh biopsies for direct SRS imaging. (**b**) It features the properties of Femto-SRS and Pico-SRS, including pulse chirping, spectral resolution and the conversion of a single Femto-SRS image into a pair of Pico-SRS images using deep U-Net. (**c**) Multi-chemical imaging of gastric tissue including lipid, protein and collagen fibers visualized through converted Femto-SRS and SHG channels, color-coded to SRH. Scale bars: 50 µm. (adopted from Liu et al. [85]).

Liu et al. compared picosecond laser-based imaging with single-shot femtosecond SRS (Femto-SRS) and demonstrated that the methodology can reach maximal speed and accuracy by integrating with U-Net [85]. As shown in Figure 2, excised tissues from gastroscopic surger, were placed upon a glass slide and analyzed with an SRS microscope. The speciality of the work was that it could generate highly chemically precise Pico-SRS from Femto-SRS. The basic difference observed between Femto- and Pico-SRS methodologies was that the former, despite having the advantages of high speed and high SNR, is compromised by its weakness in chemical selectivity. While Femto-SRS casts single-shot single channel images, Pico-SRS takes raw images at two Raman frequencies (ω_1_ = 2845 cm^−1^ for CH_2_, ω_2_ = 2930 cm^−1^ for CH_3_) to extract lipid/protein distributions with very high compositional precision. An engineered U-Net was designed to take in the single channel Femto-SRS and split it up into a dual channel Pico-SRS, eliminating the need for complicated optical engineering or physical tuning of detection frequencies, thereby doubling the imaging speed at half of the laser power, while preserving the spatio-chemical information of the image. The U-Net consisted of convolutional layers with five down-sampling layers and five up-sampling layers and a pooling layer in each. Conversion efficiency was confirmed by cross-comparing with originally recorded Pico-SRS images using the same FOV (Figure 2b). As an aid, SHG was used to image collagen fibers. Multi-chemical imaging results of gastric tissues composed of lipid (green), protein (blue) and collagen fibers (red) from the output SRH can be observed in Figure 2c. The originally acquired picosecond SRS images were used as the ground truth for training the U-Net. After U-Net processing into dual-channel SRS images, chemical decomposition was implemented to output images of proteins and lipids by simple linear algebra, and collagen images were sourced from SHG.

**Figure 3:**
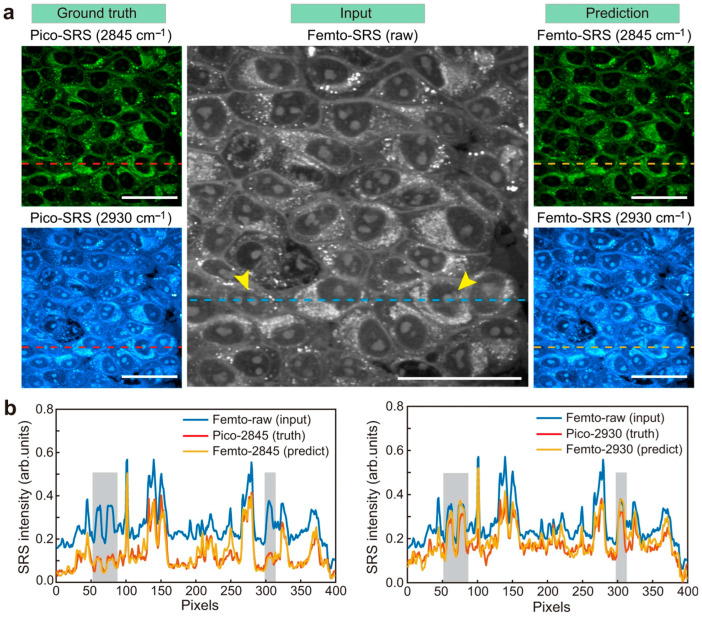
(**a**) Deep U-Net based Femto-SRS imaging originally acquired Pico-SRS images of two channels (ground truth): the single channel Femto-SRS raw image (input), and the U-Net based prediction. Scale bars: 50 µm. (**b**) Intensity profiles corresponding to the dashed lines in (**a**) of the predicted and ground-truth data, showing chemical contrast in the cell nucleus regions (marked in yellow arrows (**a**) and in grey (**b**). (Source Liu et al. [85]).

The application of this methodology can be observed in Figure 3a, where the originally acquired Pico-SRS at two different Raman shifts (ground truth), a conventional Femto-SRS raw image (input), and the results obtained from the U-Net-mediated conversion of Femto-SRS to a dual channel Pico-SRS (prediction) are presented. It can be observed that the U-Net image closely matches with the ground truth image, especially in the 2845 cm^−1^. The intensity profiles of the dashed line for ground truth corresponding to the f cell nucleus region can be observed in Figure 3b. It can also be observed that the peak intensity profiles of ground truth and prediction match very closely, with slight variation in the 2845 cm^−1^ regions, especially in the shaded region used for image generation. Overall results indicate that the U-Net methodology is extremely capable of realistically converting the single channel Pico-SRS into dual channel Femto-SRS.

**Figure 4:**
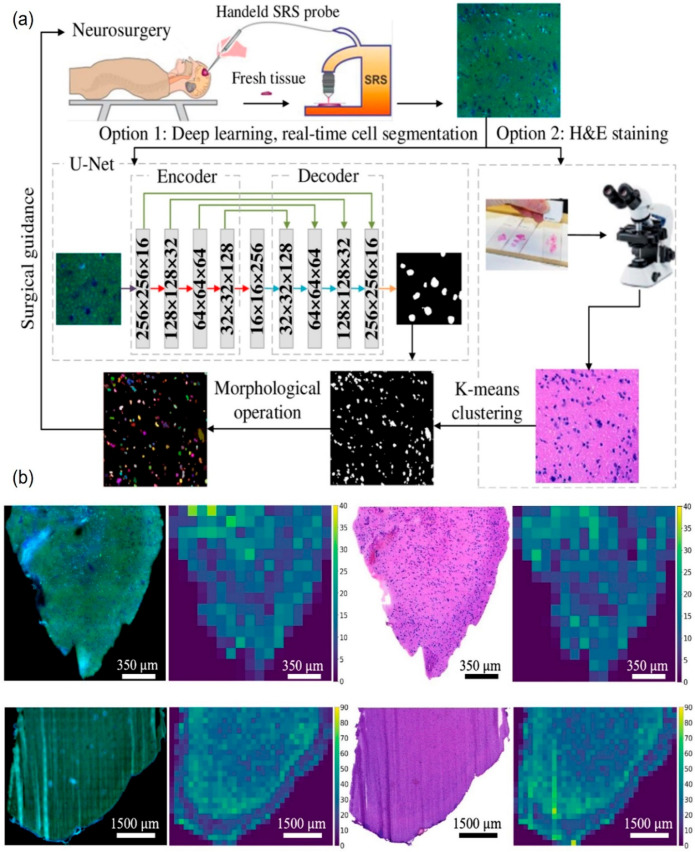
(**a**) The process flow of the SRH analysis. The tissue extracted during excision surgery was analyzed using SRH and H&E analysis. SRH image analysis was performed using U-Net as option 1. Option 2 performs H&E staining and subsequent analysis using K-means clustering. The outputs from option 1 and option 2 were subjected to morphological operation. (**b**) Cell segmentation and identification results in a FOV, where the number of cells for each patch is mapped to visualize cell distribution within a sample (Adopted from Zhang et al. [103]).

Furthermore, the team utilized these two CNNs to perform semantic segmentation and derive a heatmap of the SRS image. Each sub-tile of size (50 × 50 px) was analyzed 36 times to generate probability maps, which were then projected onto the Femto-SRS image to generate the heatmap denoting intratumor heterogeneity and possible resection margins. In the first instance of deep learning-based cell counting on SRS images, Zhang et al. developed a split and combine method wherein the U-Net is adapted to efficiently perform cell segmentation and cell counting from brain tumor SRS images [103]. The conventional U-Net architecture is altered and tailored to segment cells across brain tumor samples, utilizing a limited set of annotations. The process flow can be observed in Figure 4, which shows the overview of the cell counting framework. This can be regarded as a hierarchical approach involving (1) cell semantic segmentation and (2) morphological operation. The first stage involves cell semantic segmentation, where there are two machine learning options: deep learning near-real-time segmentation with U-Net, and K-means clustering. The second stage of morphological analysis involves distance transform and watershed segmentation algorithms, which recognize distinct cell instances.

As can be seen, excised tissue samples are extracted intraoperatively and are analyzed using SRS setup. The tissue samples are subjected to U-Net-based split and combine method analysis. The architecture of the U-Net model can be observed in Figure 4a. Cell segmentation involves cropping images into small patches of size 256 × 256 pixels and later combining the segmented patch results, which results in dependable analysis. The U-Net architecture includes an encoder and decoder, where the encoder implements the convolutional process, and decoder applies the deconvolutional process. The U-Net architecture also included 16, 32, 64, 128, and 256 kernels for the encoder and decoder in the five levels, which reduces the model’s complexity and the number of parameters to be optimized. Option 1 involves analyzing the SRH images using the deep learning, real-time U-Net cell segmentation method. For option 2, the study utilized the K-means clustering method, which involves a H&E staining process, making it time-consuming and hence impractical as a quick diagnostic methodology. The K-means clustering methodology is an unsupervised learning algorithm that has the advantages of efficient calculation and better understanding. The objective function of the samples X = {x_1_, …, x_n_} used for clustering samples with K clusters is

$$J(C)=\sum_{k=1}^{k}\sum_{x_{i}\in C_{k}}{\left\|x_{i}-{µ}_{k}\right\|}^{2}$$

where x_i_ − µ_k_ is the similarity between x_i_ and µ_k_, and µ_k_ is the centroid of cluster k. The similarity measure selected is Euclidean distance. The paired H&E brain sample images served as a reference to assess the cell counting results using the SRS images. H&E images were also analyzed in parallel via the clustering method, which groups the pixels from the H&E image into groups by recognizing statistically comparable clusters.

Cell counting was performed on SRS and H&E images using option 1 and option 2, respectively, as shown in Figure 4. Furthermore, a morphological opening operation was employed to reduce noise, and associated regions are labeled as initial cell instances using the OpenCV toolbox. However, the presence of overlapping cells affects the precision of the counting; to address this, a post-morphological analysis that uses distance transform and watershed segmentation algorithms was used for each region. The distance transform algorithm creates a distance map among the pixels of each cell image, standardizing it to determine a threshold value that distinguishes between the cell and background. Any pixels falling within the ridgeline are further processed by the watershed algorithm, which works by tracing all pixels toward a local minimum in the direction of steepest descent, which helps to group the pixels according to the paths to a cell instance. This combination of methodologies yielded high efficiency in cell instance segmentation and cell counting. Finally, suppression of noise is attained by excluding regions smaller than 0.37 µm and removing strong protein or lipid signals that generate noise.

The team further compared various U-NET configurations like U-Net, 7 layer-U-Net, 5 layer-U-Net, FCN and the modified U-Net, of which the modified U-Net reported the best efficiency. Along with the parameters of accuracy, AUC, sensitivity and specificity, DICE coefficient and percentage error were included. DICE coefficient is used to evaluate the spatial overlap of models and percentage error is used to evaluate the performance of cell counting. In cell counting using SRS images of real human brain tumor specimens, results were obtained with >98% AUC and R = 0.97 in comparison with H&E staining. The study illustrates the immense potential of SRS to be used as a modality for pathology analysis and cell counting in near-real time. However, the necessity of U-Net for manually generated cell annotation often exposes the model to subjective errors and weak cell contrast. Despite the implementation of the watershed segmentation algorithm, all overlapped cells cannot be split, which also poses challenges to absolute accuracy in cell counting.

In 2020, Hollon et al. reported a study that utilized CNNs, trained over 2.5 million SRH images, capable of intraoperatively diagnosing brain tumors in under 150 s, which is far better than conventional methods, which take up to 30 min [104]. The study was a multicenter clinical trial with 278 participants, and CNN-based diagnoses attained 94.6% accuracy in comparison with 93.9% attained by conventional methods. To identify the features learned by the CNN for each class, a methodology called maximal mean activation was employed. This methodology refers to the highest average activation value of the neurons in a particular layer of a CNN for a given class, by engaging gradient ascent in the input space. It was revealed that the deep hidden layers detected nuclear and chromatin morphology, axonal density and histoarchitecture as domain-specific features. The images generated through activation maximization produced recognizable features for each histologic class, such as lipid-rich axons in gray matter and, in the case of malignant glioma, high nuclear density, lipid droplets, and features associated with higher-grade gliomas. In the case of metastatic tumor cells and pyramidal neurons, cytoplasmic vesicles and large nuclei with prominent nucleoli were observed, indicating that the CNN had learned importance of specific histomorphologic, nuclear and cytologic features for image classifications. The methodology also attempted semantic segmentation of SRH images to identify tumor-infiltrated diagnostic regions within SRH images.

**Figure 5:**
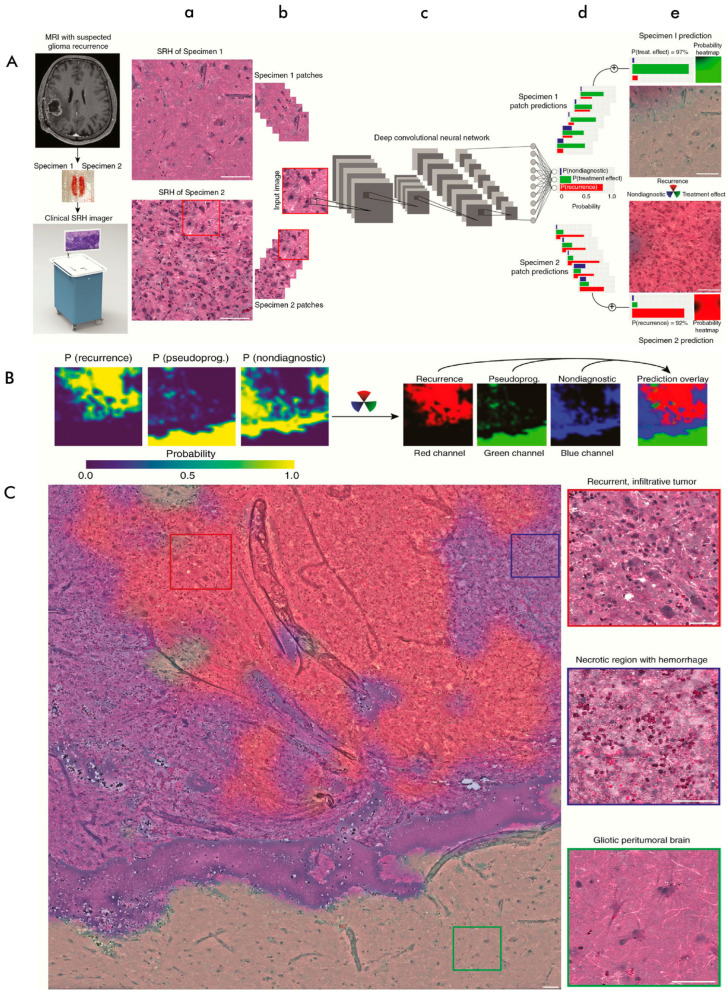
(**A**) (**a**–**d**) The SRH and CNN workflow for the automated detection of recurrent glioma. (**a**) A 1 × 1-mm SRH image is captured in about 60 s, (**b**) which gets split into 300 × 300-pixel patches using a dense sliding window method. (**c**) Each patch is analyzed by a feedforward CNN. (**d**) The final softmax layer produces a categorical probability distribution across classes: recurrence, pseudo-progression/treatment effect, and nondiagnostic. (**e**) An aggregation algorithm aggregates patch-level prediction probabilities to yield a single probability of recurrence for each specimen or patient. Scale bars = 50 μm. (**B**) Probability heatmaps for each of the three output classes are generated using patch-level predictions obtained from a dense, overlapping sliding window algorithm. This method ensures that each pixel in the image has a corresponding probability distribution, resulting in high-resolution, smoother heatmaps. (**C**) Each heatmap is assigned to an RGB channel, producing an overlay of predictions on the entire SRH slide. An SRH image from a patient with recurrent glioblastoma is shown, where dense tumor areas (red) are highlighted alongside nondiagnostic regions such as hemorrhagic and necrotic tissue (blue) and gliotic brain tissue (green). This semantic segmentation technique enhances the interpretation of SRH images by combining CNN predictions with spatial information about recurrent tumor areas. Scale bars = 50 μm (adopted from Hollon et al. [91]).

In 2021, Hollon et al. reported a study that was aimed at exploiting the possibilities of using CNNs to create an inference algorithm for identifying viable recurrent glioma [91]. The 300 × 300-pixel sliding window algorithm was employed, rolling at a 100-pixel step size to generate patches from SRH images. As can be observed from Figure 5, the sliding methodology yielded high-resolution, high-magnification patches, resulting in a substantially large dataset. Generated patches were rescaled and readjusted for contrast by trimming the top and bottom 3% of pixels (based on intensity) from individual channels. Subsequently, CNN employed Inception-ResNet-v2 architecture for classification, as shown in Figure 5A. Pre-training of the CNN model was conducted on approximately 3.5 million SRH images representing 14 histologic subtypes. For data augmentation, affine transformations such as rotation, shift, and reflection were applied. After pre-training, convolutional layers were retained while the final classification layers were modified for classification into three diagnostic classes: tumor recurrence, pseudo-progression, and nondiagnostic tissue. Subsequently, the network was trained for a fixed number of epochs for each fold using 406,800 patches from 35 patients; training was performed without hyperparameter tuning to prevent overfitting to the validation set. This was followed by five iterations of k-fold cross-validation, resulting in five predictions for each patient. The AI model, based on the Inception-ResNet-v2 CNN architecture, classified image patches sized 300 × 300 pixels. The best-performing CNN was selected based on cross-validation and subjected to external validation on a separate testing set comprising 48 patients.

To integrate the patch-based classifications into an aggregated CNN prediction of a single specimen, a method termed as an inference algorithm had to be developed. The CNN softmax product for each SRH patch gives a distribution of probability across the diagnostic classes. All such individual patch diagnoses from the sample were consolidated, non-diagnostic patches were eliminated, and the softmax vectors were added elementwise to generate an unnormalized probability distribution over the entire specimen. Based on the diagnostic threshold calculated from ROC, a final diagnosis can be attained. The semantic segmentation technique was tailored for SRH images to portion regions of tumor recurrence, pseudo-progression, and nondiagnostic areas. The team also enforced a semantic segmentation method that covers SRH-CNN probability heatmaps to determine the spatial regions of tumor recurrence or pseudo-progression.

Hollon et al. produced another study in which they developed an AI model named ‘DeepGlioma’ which attained 93.2% accuracy in molecular classification [105]. DeepGlioma is a deep neural network-based AI model and is designed to predict paramount genetic changes in diagnosing diffuse glioma. It achieves molecular classification within two minutes, avoiding the need for human interpretation. The AI workflow started when, after acquiring SRH images, the molecular classification model was trained with a multimodal approach on two datasets: clinical SRH images and genomic sequencing data. This work identified that weakly supervised patch-based contrastive learning (patchcon) was ideal for whole-slide SRH classification. The team formed a simple and general framework for multi-label contrastive learning of visual representations and trained the SRH encoder using this framework. A genetic embedding model (with which gene information is effectively represented as numerical vectors), inspired by joint semantic-visual embedding space and text-to-image generation methodologies, was pre-trained using large-scale, public glioma genomic data. The co-accompaniment of specific mutations in the same tumor type portrays the molecular subgroup of diffuse gliomas. These co-occurrences were effectively learned by the model using global vector embeddings, and the training strategy learned a linear substructure that matches known molecular subgroups of diffuse gliomas. Afterwards, the pre-trained SRH and genetic encoders are combined within a unified transformer model for multi-label molecular classification. Masked labeling, where a group of genes were masked during input was also employed during training to leverage the advantages of genetic encoder pre-training and the learned substructure of molecular subgroups, wherein the transformer output acts as the pre-trained embedding space. To display the advantages of patchcon, genetic pre-training, and masked label transformer training, the team conducted iterative hold-out cross-validation. Subsequently, to strictly evaluate the model, a series of leave-institution-out cross-validation (LIOCV) trials were conducted to assess DeepGlioma’s stability across multiple medical centers involved in the study, and to investigate the impact of increased training data on model performance. To sum up, the study developed a transformer-based multi modal training strategy that uses a pre-trained SRH image feature encoder and a large-scale genetic embedding model to achieve optimal molecular classification performance, accomplishing a genetic classification accuracy of 93.2% and accurately identifying the diffuse glioma molecular subgroup with 91.5% precision. The developed methodology was further evaluated in a prospective international study. The leave-institution-out cross-validation yielded stable performance, with a molecular classification accuracy standard deviation range of ±2.75–6.06% and an F1 score range of ±1.71–4.70%.

Attempting to accelerate predictions of intraoperative tumor presence, Reinecke et al. devised a novel AI model featuring a deep residual CNN with an automated pipeline [106]. In a monocentric prospective clinical study conducted with 94 patients undergoing biopsy or resection of brain or spinal tumors, intraoperative tissue samples were imaged using a fiber-laser-based SRS microscope to obtain SRH images. A ResNetV50 residual network was orchestrated and trained to classify three classes of images as tumor, nontumor and low-quality. The network was trained on a separate previously acquired and annotated dataset of 570 whole-slide SRH images that result in 1.2 million labeled patches (300 × 300 px) after patch extraction. The CNN training/validation ratio was maintained at 90:10. Class imbalance of the dataset was mitigated utilizing inverse class frequencies as weights for the categorical cross-entropy loss function of the CNN. Training continued until the accuracy surpassed 95% and the loss fell below 0.10. Three CNN models were separately trained and evaluated using different random seeds on the same dataset. The CNNs yielded tumor mean probabilities of 73.9 (±33.2), 76.9 (±35.0) and 76.5 (±33.7), respectively. Inter-rater analysis of probability values among the three CNNs demonstrated excellent reliability, with an ICC value of 0.962 (99% CI 0.953–0.969). Similarly, the mean probabilities for the non-tumor class were 18.9 (±33.1), 18.0 (±32.7) and 18.2 (±31.6), with an ICC value of 0.977 (99% CI 0.973–0.981) between the CNNs. For the low-quality output class, the mean probabilities were 7.2 (±15.2), 5.1 (±16.4) and 5.3 (±15.7) for the three CNNs, respectively, with an ICC of 0.914 (99% CI 0.895–0.929) indicating excellent inter-rater reliability. The formulated residual network was evaluated by analyzing images from three randomly selected areas within the tissue samples in comparison with neuropathologists’ observations.

## 4. Conclusions and Perspectives

Exponentially growing rates of diseases like cancer, along with a worldwide scarcity of pathologists, have created an acute need for devices that can swiftly characterize tissues with high precision in a label-free manner without the need of staining, which can often alter the integrity of the sample. SRH has evolved over years, leveraging the recent advancements in fiber-laser, hardware and optical fields, to be a prominent alternative based on vibrational imaging modalities. This innovative, non-linear technique exploits the principles of Raman spectroscopy to render comprehensive molecular information about tissue analytes. The methodology has already been able to deliver reliable-quality imaging that is helpful for precise diagnosis within reasonable time frames and continues to improve with advancements in technology, AI/ML, multiplex tags, probes, etc. This laser-based modality holds potential to be incorporated into microscopes, biopsy needles and point probes, which extends its usefulness as a diagnostic device. This can further be combined with other useful modalities like SHG, TPEF, etc., which can aid histologic imaging processes and reliable decision making. In the context of stimulated Raman histology (SRH), the balance between Raman bandwidth and imaging speed is a crucial aspect of achieving high-quality images while maintaining practicality for biological tissue analysis. To attain practically feasible timelines, the current system confines the analysis to lipid- and protein-representing Raman peaks. If a wider bandwidth is used, the imaging process might become slower as more data points are collected. However, narrowing the bandwidth may speed up the process but lose important information about the biochemical makeup of the tissue, which may be very crucial in representing the biochemical profile of the analyte. Simultaneously improving Raman bandwidth and imaging speed may be attained by engaging recent advancements in multiplexed Raman, while fast spectral acquisition techniques can facilitate higher-speed acquisition with more spectral data. Such an improvement can help to overcome the biggest challenge of SRH, especially in the context of broader organ application, i.e., overdependence on lipid-protein contrast. This lipid-protein contrast is not dependable in all contexts, and new recording color schemes must be employed to reflect more molecular compositions. These advancements should aim to record high-quality signals while keeping both speed and bandwidth in an optimal range for histological analysis.

It is also worth noting that there is scope for improvement in a few other aspects, such as automated tissue handling during analysis. Currently, tissue samples for analysis should be of uniform consistency and size, which permits uniform compression over the glass slides, and be positioned well within the focal area of the excitation laser. Stiffer tissues and those greater than 3 mm in size can affect the imaging quality, as they may fall outside the field of vision of the device, necessitating multiple sessions of imaging. These aspects may be addressed by incorporating an AI-based surface profiling methodology, which can map the tissue for consistency, texture and geometry beforehand. Such automated handling can retain the imaging surface sharply within the laser focal area to perform optimal imaging. It is also worth noting that, even though SRH imaging closely resembles conventional H&E staining, the matches between both methodologies are not exact and require some expertise for histopathologists to analyze them.

Recent advancements in AI/ML-based image analysis have tremendously helped the modality in shortening time frames while attaining high precision. Several studies have established the effectiveness of the modality in histologic purposes, most of which have been summarized in this review. The blend of AI and SRH has huge potential and can safely be considered as a next-generation imaging tool that can aid and relieve the workload for pathologists.
